# N-Cadherin Relocalizes from the Periphery to the Center of the Synapse after Transient Synaptic Stimulation in Hippocampal Neurons

**DOI:** 10.1371/journal.pone.0079679

**Published:** 2013-11-01

**Authors:** Patricia T. Yam, Zachary Pincus, Gagan D. Gupta, Mikhail Bashkurov, Frédéric Charron, Laurence Pelletier, David R. Colman

**Affiliations:** 1 Department of Neurology and Neurosurgery, Montreal Neurological Institute and Hospital, McGill University, Montreal, Quebec, Canada; 2 Program in Neuroengineering, McGill University, Montreal, Quebec, Canada; 3 Department of Molecular, Cellular and Developmental Biology, Yale University, New Haven, Connecticut, United States of America; 4 Samuel Lunenfeld Research Institute, Mount Sinai Hospital, Toronto, Ontario, Canada; 5 Molecular Biology of Neural Development, Institut de Recherches Cliniques de Montréal (IRCM), Montreal, Quebec, Canada; 6 Department of Medicine, University of Montreal, Montreal, Quebec, Canada; 7 Department of Anatomy and Cell Biology, Department of Biology, Division of Experimental Medicine, McGill University, Montreal, Quebec, Canada; 8 Department of Molecular Genetics, University of Toronto, Toronto, Ontario, Canada; Virginia Tech Carilion Research Institute, United States of America

## Abstract

N-cadherin is a cell adhesion molecule which is enriched at synapses. Binding of N-cadherin molecules to each other across the synaptic cleft has been postulated to stabilize adhesion between the presynaptic bouton and the postsynaptic terminal. N-cadherin is also required for activity-induced changes at synapses, including hippocampal long term potentiation and activity-induced spine expansion and stabilization. We hypothesized that these activity-dependent changes might involve changes in N-cadherin localization within synapses. To determine whether synaptic activity changes the localization of N-cadherin, we used structured illumination microscopy, a super-resolution approach which overcomes the conventional resolution limits of light microscopy, to visualize the localization of N-cadherin within synapses of hippocampal neurons. We found that synaptic N-cadherin exhibits a spectrum of localization patterns, ranging from puncta at the periphery of the synapse adjacent to the active zone to an even distribution along the synaptic cleft. Furthermore, the N-cadherin localization pattern within synapses changes during KCl depolarization and after transient synaptic stimulation. During KCl depolarization, N-cadherin relocalizes away from the central region of the synaptic cleft to the periphery of the synapse. In contrast, after transient synaptic stimulation with KCl followed by a period of rest in normal media, fewer synapses have N-cadherin present as puncta at the periphery and more synapses have N-cadherin present more centrally and uniformly along the synapse compared to unstimulated cells. This indicates that transient synaptic stimulation modulates N-cadherin localization within the synapse. These results bring new information to the structural organization and activity-induced changes occurring at synapses, and suggest that N-cadherin relocalization may contribute to activity dependent changes at synapses.

## Introduction

Cadherins are Ca^2+^ dependent homophilic adhesion molecules. In the nervous system they are involved in neural tube formation, stratification of cells into layers, tissue coherence and laminar targeting [1,2]. One widely distributed neural cadherin is N-cadherin, a single pass transmembrane protein composed of five extracellular cadherin repeat domains, a single membrane spanning region, and a conserved intracellular domain [2]. Strong intercellular adhesion results from the concerted binding of many cadherin molecules from apposing membranes, and is thought to require both trans homodimerization and lateral clustering through cis interactions [3]. Intracellularly, N-cadherin is linked to the actin cytoskeleton through catenin proteins (β-catenin, p120catenin/δ-catenin) binding to its cytoplasmic domain [3,4]. 

N-cadherin is enriched at synapses, a specialized asymmetric cell-cell junction that mediates neuronal communication [5,6]. At the synapse, N-cadherin connects the pre- and post-synaptic membranes and also influences synaptic morphology and function. For example, presynaptically, N-cadherin regulates synaptic vesicle recruitment and recycling [7,8], and this depends at least in part on the association of N-cadherin with β-catenin [9]. Postsynaptically, inhibition of N-cadherin function results in thinner and filopodium-like spines, whereas increased N-cadherin expression results in larger and more mature spines [8,10,11], implicating N-cadherin in spine remodelling. N-cadherin also regulates the trafficking of AMPA receptors [12,13]. Interestingly, N-cadherin is involved in synaptic potentiation and plasticity, the cell biological processes thought to underlie learning and memory. N-cadherin is required for activity-induced spine expansion and stabilization [10,14,15] and N-cadherin is required for hippocampal LTP [14,16,17]. Furthermore, post-synaptic N-cadherin can regulate pre-synaptic organization and transmitter release [18] and pre-synaptic short-term synaptic plasticity [19].

N-cadherin itself is also modulated by synaptic activity. Synaptic stimulation increases N-cadherin dimer formation and resistance to trypsin digestion [20]. N-cadherin endocytosis is regulated by NMDAR activation and reciprocally, stabilization of surface N-cadherin blocks short term NMDAR-dependent synaptic plasticity [21]. N-cadherin endoyctosis is also regulated by the protocadherin arcadlin after synaptic stimulation [22]. These activity dependent changes in N-cadherin endocytosis may be involved in linking synaptic activity with N-cadherin localization and spine morphology [21,22]. 

The functional and structural roles of cadherin are likely to be interlinked, and we hypothesized that synaptic activity could change the localization of N-cadherin within synapses, and this could be one mechanism via which N-cadherin regulates synaptic strength and stability. N-cadherin has been shown to localize in clusters adjacent to the active zone [23,24], which may correspond to puncta adherentia [25]. Alternatively, N-cadherin has been reported to form a ring surrounding the active zone [6] and postulated to act as a gasket encircling the active zone, possibly containing the secretion of neurotransmitters [26]. To directly visualize the subsynaptic localization of N-cadherin in a large population of synapses, we used structured illumination microscopy (SIM), a super-resolution microscopy method. We found that in contrast to a defined ring structure or simple cluster, N-cadherin localizes in a spectrum of patterns within the synapse, from clusters adjacent to the active zone to an even distribution along the active zone, with variations in between these extremes. Furthermore, synaptic stimulation converts N-cadherin localization from a peripheral to a more central distribution within the synapse.

## Results and Discussion

### Structured illumination microscopy can resolve different synaptic domains

**Figure 1:**
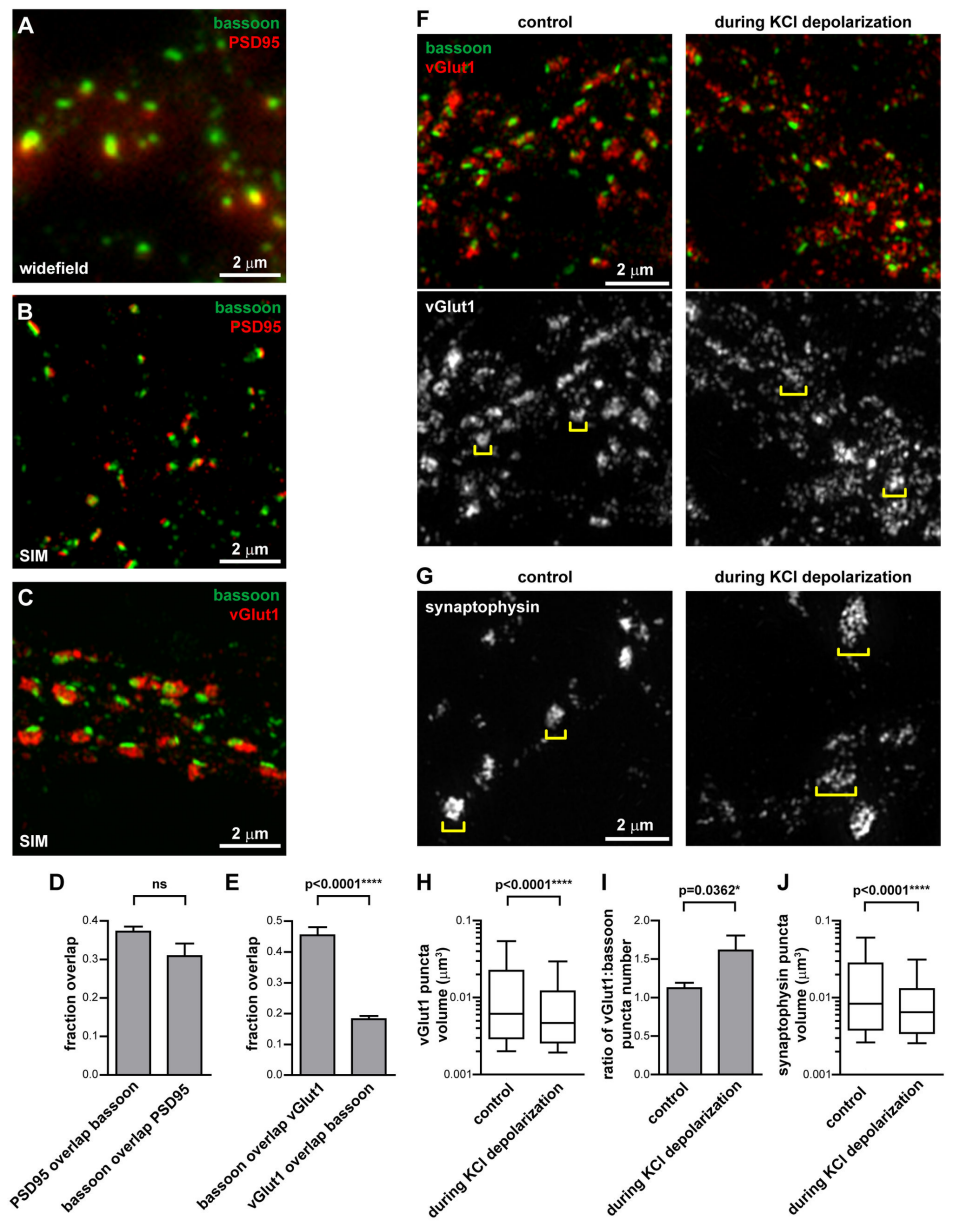
Structured illumination microscopy can resolve different synaptic domains. (**A**,**B**) 17-20 DIV hippocampal neurons immunostained for the active zone protein bassoon and the post-synaptic density protein PSD95, or (**C**) bassoon and the synaptic vesicle protein vGlut1. (**A**) Imaged with widefield fluorescence microscopy, bassoon and PSD95 puncta overlap. (**B**) Imaged with SIM, the increased resolution shows that bassoon and PSD95 occupy separate domains, consistent with their function in the pre- and post-synaptic compartments respectively. (**C**) Within the presynaptic bouton, the synaptic vesicle pool (represented by vGlut1) and the active zone (represented by bassoon) can be distinguished with SIM. (**D**) Manders’ coefficient measurement of the proportion of PSD95 signal which overlaps with the bassoon signal and vice versa. Unpaired t-test, 6 fields of view. (**E**) Manders’ coefficient measurement of the proportion of bassoon signal which overlaps with the vGlut1 signal and vice versa. Unpaired t-test, 6 fields of view. Graphs represent the mean ± s.e.m. (**F**,**G**) During depolarization of neurons with 50 mM KCl, the synaptic vesicle markers vGlut1 and synaptophysin become smaller and more numerous, and spread over larger area (brackets). (**H**) The vGlut1 puncta volume decreases during KCl depolarization. Box and whiskers graph. Mann Whitney test, 7 fields of view, ≥980 vGlut1 puncta analyzed per condition. (**I**) The number of vGlut1 puncta per bassoon puncta increases during KCl depolarization. Graph represents the mean ± s.e.m. Unpaired t-test, 7 fields of view, ≥874 bassoon puncta analyzed per condition. (**J**) The synaptophysin puncta volume decreases during KCl depolarization. Box and whiskers graph. Mann Whitney test, ≥10 fields of view per condition, ≥533 synaptophysin puncta analyzed per condition.

Conventional light microscopy has a resolution limit of ~200-250 nm, which is insufficient to resolve details within synapses which generally range in diameter from ~200-500 nm. Thus to visualize how N-cadherin localizes within synapses, we turned to SIM, a super-resolution microscopy technique with a two-fold greater resolution along the lateral and axial directions, resulting in an x-y resolution of ~100 nm [27–29]. We studied synapses in cultured rat hippocampal neurons at 17-20 DIV (days in vitro), by which time mature synapses were present. Immunostaining these neurons for bassoon, an active zone (presynaptic) protein, and PSD95, a postsynaptic density protein, revealed mostly overlapping puncta when imaged with conventional widefield fluorescence microscopy (Figure 1A). When imaged with SIM, the bassoon and PSD95 puncta were more clearly resolved and occupied adjacent domains apposed to each other, reflecting their localization to opposing sides of the synapse (Figure 1B). We measured the overlap between the bassoon and PSD95 signals with the Manders’ coefficient [30], which indicates the fraction of the bassoon signal coincident with the PSD95 signal and vice versa. This showed that only one-third of the signal between the bassoon and PSD95 stainings overlapped (Figure 1D), implying that most of the bassoon and PSD95 signals occupy different zones. Given that bassoon and PSD95 on either side of the synaptic cleft are separated by ~90 nm [31], which is less than the x-y resolution of SIM at ~100 nm, and that synapses are not always oriented with their pre- and post-synaptic domains aligned along the x-y plane, some colocalization between the bassoon and PSD95 signal would be expected. However, most of the bassoon and PSD95 signal are not colocalized, indicating that when imaged with SIM, the bassoon and PSD95 puncta are distinct, slightly overlapping domains. 

We next immunostained and imaged with SIM two proteins within the presynaptic compartment - bassoon and vGlut1. The size of the bassoon puncta when co-immunostained with vGlut1 (Figure 1C) was the same as when co-immuostained with PSD95 (Figure S1). Imaging with SIM showed that bassoon and vGlut1 segregated into different zones (Figure 1C), consistent with their known subsynaptic localization to the active zone and to the synaptic vesicle pool respectively [32]. We analyzed the proportion of the bassoon signal coincident with the vGlut signal and vice versa, and found that 45% of the bassoon colocalized with vGlut1, but only 18% of the vGlut1 colocalized with bassoon (Figure 1E). This is consistent with the vGlut puncta occupying a much larger area than the bassoon puncta (Figure 1C) and only colocalizing with bassoon near the active zone. Away from the active zone in the pre-synaptic bouton, there is vGlut1 present in the absence of bassoon [32].

Knowing that SIM could resolve different synaptic domains, we next determined whether it was sensitive enough to visualize changes in these domains. In unstimulated neurons, vGlut1 and synaptophysin, synaptic vesicle membrane proteins, formed tight clusters adjacent to the active zone (Figure 1F,G). During depolarization of neurons with KCl, vGlut1 and synaptophysin dispersed and spread out over a larger area (Figure 1F,G). We measured the dispersion of the vGlut1 and synaptophysin staining into smaller and more numerous puncta in two ways. Firstly, the volume of vGlut1 and synaptophysin puncta decreased during depolarization (Figure 1H,J). Secondly the number of vGlut1 puncta per bassoon puncta increased during depolarization (Figure 1I). This dispersion of synaptic vesicle markers is consistent with depolarization inducing synaptic vesicle fusion with the presynaptic membrane, leading to expansion of the presynaptic membrane [33] and dispersion of synaptic vesicle proteins along the presynaptic membrane.

### N-cadherin has a spectrum of distributions at the synaptic cleft

**Figure 2:**
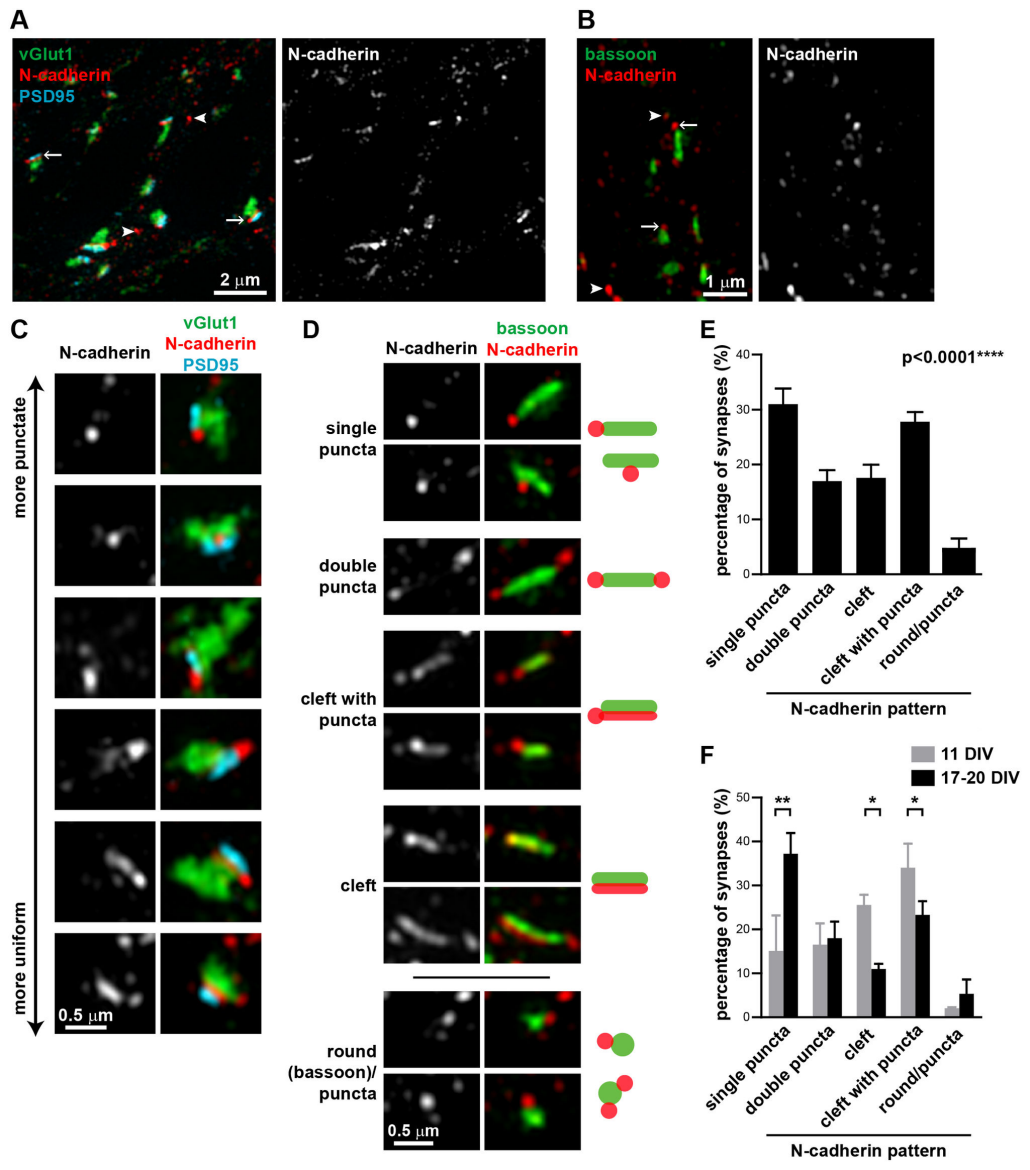
N-cadherin has a spectrum of localization patterns at the synaptic cleft. (**A**) N-cadherin is localized between the pre- and post-synaptic compartments, represented by vGlut1 and PSD95 respectively. (**B**) N-cadherin is localized at or adjacent to the active zone, represented by bassoon. *Arrows*, N-cadherin puncta associated with synapses; *arrowheads*, N-cadherin puncta not associated with synapses. (**C**) N-cadherin localization at synapses varies from punctate, often flanking one side of the synapse, to uniform along the synaptic cleft. (**D**) Classification of N-cadherin localization patterns relative to bassoon into five categories (single puncta, double puncta, cleft with puncta, cleft, and round(bassoon)/puncta). Representative images and schematics of the five different N-cadherin localization patterns. (**E**) Percentage of synapses (mean±s.e.m) in 17-20 DIV hippocampal neurons in each N-cadherin pattern category. n=7 experiments, ≥77 synapses per experiment, 650 synapses total. p<0.0001, one-way ANOVA, Tukey’s post-test (p<0.01 for single puncta vs. double puncta; p<0.01 for single puncta vs. cleft; p>0.05 for single puncta vs. cleft with puncta; p<0.001 for single puncta vs. round/puncta). (**F**) Percentage of synapses (mean±s.e.m) in each N-cadherin pattern category in 11 DIV hippocampal neurons compared to matched cultures at 17-20 DIV. N-cadherin at synapses from neurons at 11 DIV is distributed more evenly along the synaptic cleft and less as puncta compared to synapses from neurons at 17-20 DIV. Two-way ANOVA with matched values and Bonferroni post-test. n=2 experiments, ≥103 synapses per experiment.

We next used SIM to visualize the localization of N-cadherin in hippocampal neurons at 17-20 DIV. N-cadherin puncta were present both at synapses (arrows) and outside of synapses (arrowheads) (Figure 2A,B). That N-cadherin has both synaptic and non-synaptic localizations has been previously observed [21,22]. The N-cadherin puncta outside of the synapses could be N-cadherin in transport vesicles [34], or have non-synaptic functions such as at adherens junctions and in axon outgrowth. At synapses, N-cadherin was localized between vGlut1 and PSD95 (Figure 2A), consistent with its role as an adhesion molecule between the pre- and post-synaptic membranes [35]. N-cadherin was also closely associated with the active zone, as defined by bassoon staining (Figure 2B) with most bassoon puncta (~97%) having N-cadherin associated with it. 

When we examined each synapse individually, we found that N-cadherin had a spectrum of localization patterns within the synapse, ranging from round puncta, which were often asymmetrically positioned on one side of the synapse, to a uniform localization along the synaptic cleft (Figure 2C). Notably, these were just two extremes of the observed N-cadherin localization patterns; it could often be a combination of a pronounced puncta at one side of the synapse with a fainter localization along the synaptic cleft. 

We quantified these N-cadherin localization patterns relative to bassoon, since it delineates the active zone parallel to the synaptic cleft and removal of N-cadherin does not affect bassoon [7], making it an appropriate reference protein. Bassoon staining at synapses typically had an elongated rod shape. We classified the N-cadherin localization pattern at the synapse with respect to bassoon into five categories (Figure 2D): (1) single puncta, which commonly flanked one end the active zone, (2) double puncta, often flanking both ends of the active zone, (3) cleft with puncta, with N-cadherin puncta flanking the active zone and fainter N-cadherin partly or fully along the synaptic cleft, (4) cleft, where the N-cadherin staining was predominantly along the synaptic cleft, with little staining outside the active zone area and (5) round(bassoon)/puncta, where the bassoon staining was more round than the typical rod-shape, with punctate N-cadherin staining associated with it. The two most common distributions were single puncta (31%) and cleft with puncta (27.8%) (Figure 2E). Slightly fewer synapses were classified as double puncta (17%) and cleft (17.6%). Notably, considering the single puncta, double puncta, and cleft with puncta all as distributions with significant N-cadherin clustering adjacent to the active zone, ~76% of synapses had N-cadherin present in a cluster(s) adjacent to the active zone, whereas only 17.6% of synapses had a more homogeneous distribution of N-cadherin evenly along the synaptic cleft. Fewer than 5% of synapses were characterized by round bassoon puncta.

We also compared the N-cadherin localization within synapses from neurons at 11 DIV, when synapses are not yet mature, to synapses from neurons at 17-20 DIV. Consistent with previous studies in hippocampal neuron cultures and in vivo hippocampus [23], we found that N-cadherin in younger synapses was more evenly distributed along the synaptic cleft, compared to those in older cultures (Figure 2F). Synapses in younger cultures had significantly fewer synapses with N-cadherin present as puncta adjacent to the active zone, and significantly more synapses with N-cadherin present along the synaptic cleft or along the synaptic cleft with some punctate staining. This difference in cadherin synaptic localization in young versus mature synapses may reflect the different roles for N-cadherin in young versus mature synapses [23,36,37]. In young synapses, which are dependent on an intact F-actin cytoskeleton, N-cadherin could have a major structural role in stabilizing the synapse [23,36,37], whereas in mature synapses, N-cadherin contributes to synaptic plasticity and the maintenance of spine structure [8,14–17,23]. Changes in N-cadherin distribution with respect to PSD95 has also been observed during synapse maturation in chick ciliary neurons, with mature synapses having well-defined clusters of N-cadherin that are associated with PSD95 [38].

Immuno-electron microscopy (EM) studies have shown that N-cadherin has a clustered distribution in mature hippocampal synapses, predominantly near the edge of the active zone [14,17,23,24]. However N-cadherin distributed along the entire synaptic cleft has also been observed by immuno-EM in mature synapses [6]. With SIM, we could image and analyze more synapses than typically practical with immuno-EM, and therefore could characterize the spectrum of N-cadherin localization patterns in a large population of synapses. Thus in addition to observing both these previously described localization patterns of synaptic N-cadherin, we could also measure their relative abundance. Furthermore, immunolabeling efficiencies for fluorescence microscopy tend to be higher than that achieved with EM [39], allowing us to observe more subtle localization patterns such as the combination of lower levels of N-cadherin along the synaptic cleft with higher levels adjacent to the active zone. 

### N-cadherin moves away from the synaptic cleft during depolarization

**Figure 3:**
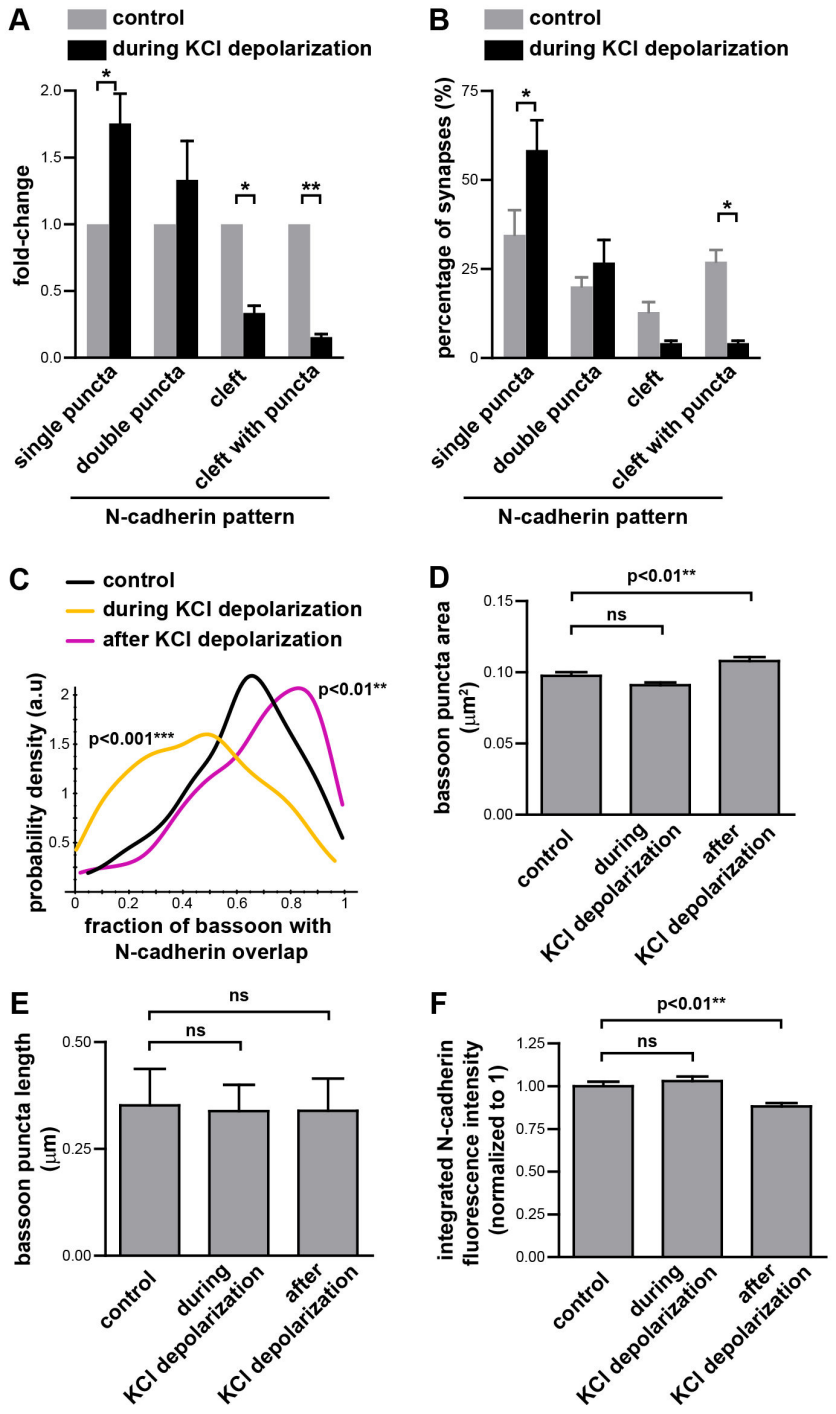
N-cadherin localization changes during and after KCl depolarization. (**A**,**B**) During depolarization with 30-50 mM KCl, the proportion of synapses with N-cadherin puncta adjacent to the active zone increases, whereas the proportion of synapses with N-cadherin along the synaptic cleft decreases. n=3 experiments, ≥88 synapses per experiment, total of 315 synapses (178 and 137 synapses for the control and during KCl stimulation conditions respectively). * = p<0.05, ** = p<0.01, two-way ANOVA with matched values and Bonferroni post-test. Graphs represent the mean ± s.e.m. (**C**) Colocalization of N-cadherin with bassoon was measured by the fraction of each bassoon puncta that overlapped with N-cadherin. The probability density function for the distribution of the values for the fraction of each bassoon puncta which overlaps with N-cadherin shows that during KCl depolarization less N-cadherin colocalizes with bassoon, whereas 15 min after transient exposure to KCl, more N-cadherin colocalizes with bassoon. Kruskal-Wallis test, Dunn’s post-test. n=3 experiments, ≥212 synapses per experiment. (**D**) The area (mean ± s.e.m) of each bassoon puncta during and 15 min after transient KCl depolarization. One-way ANOVA, Dunnett’s post-test. n=3 experiments, n≥220 synapses per experiment, 1133 synapses total. (**E**) The length of each bassoon puncta during and 15 min after transient KCl depolarization. Graph represents the median and interquartile range. Kruskal-Wallis test, p =0.1213. n=3 experiments, n≥314 synapses per experiment, 1054 synapses total. (**F**) The integrated N-cadherin fluorescence intensity of N-cadherin at each synapse was measured and normalized to the mean value of the control synapses in their respective experiment. Graph represents the mean ± s.e.m. One-way ANOVA, Dunnett’s post-test. n=3 experiments, n≥214 synapses per experiment, 1037 synapses total.

When imaged with conventional microscopy, the area of synaptic N-cadherin staining increases during depolarization [20]. To determine if this corresponds to a change in N-cadherin localization within synapses, we depolarized neurons with KCl, immunostained for N-cadherin and bassoon, and imaged with SIM. Classification of the N-cadherin localization pattern relative to bassoon showed that there was a dramatic decrease in the proportion of synapses with N-cadherin present along the cleft, and an increase in the proportion of synapses with single or double N-cadherin puncta adjacent to the active zone (Figure 3A,B). To estimate the presence of N-cadherin at the active zone, we measured the fraction of each bassoon rod that colocalized with N-cadherin. During KCl depolarization, there was a significant reduction in N-cadherin colocalizing with bassoon (Figure 3C) consistent with the decreased proportion of synapses with N-cadherin present along the synaptic cleft (Figure 3A,B). Thus the apparent increase in the area of synaptic N-cadherin staining previously observed [20] is probably due to movement of N-cadherin away from the active zone towards the periphery of the synapse, which is not resolved by conventional microscopy (widefield or confocal). We did not detect any changes in bassoon staining during KCl depolarization. Both the bassoon puncta area (Figure 1D) and the length of the bassoon puncta (Figure 1E) did not change during KCl depolarization, suggesting that the active zone itself was not grossly affected during KCl depolarization. 

To investigate whether there was also a change in the amount of N-cadherin at the synapse, we calculated the integrated fluorescence intensity of the N-cadherin signal at each synapse. We found that during KCl depolarization, the integrated fluorescence intensity of N-cadherin at synapses is the same as that of the control (Figure 3F). Hence the changes in N-cadherin localization during KCl depolarization most likely reflect translocation of N-cadherin within the synapse than net movement of N-cadherin to/from the synapse, with the movement of N-cadherin away from the active zone being consistent with depolarization inducing synaptic vesicle fusion at the active zone, adding new membrane that lacks N-cadherin. 

### Transient synaptic stimulation redistributes N-cadherin from the periphery to the center of the synapse

To test whether synaptic stimulation leads to changes in the distribution of N-cadherin within the synapse which persist after the stimulation has ended, we potentiated synapses by transient KCl depolarization, which has been shown to induce spine head expansion [15], N-cadherin dimer formation and resistance to trypsin digestion [20] and movement of β-catenin into spines [40]. Under our conditions of transient KCl depolarization followed by 15 min rest in normal media, the rod-shaped bassoon puncta were slightly (~11%) larger (Figure 3D). However, the bassoon puncta length remained the same suggesting that the small change in area was not accompanied by a change in the length of the active zone (Figure 3E). 

**Figure 4:**
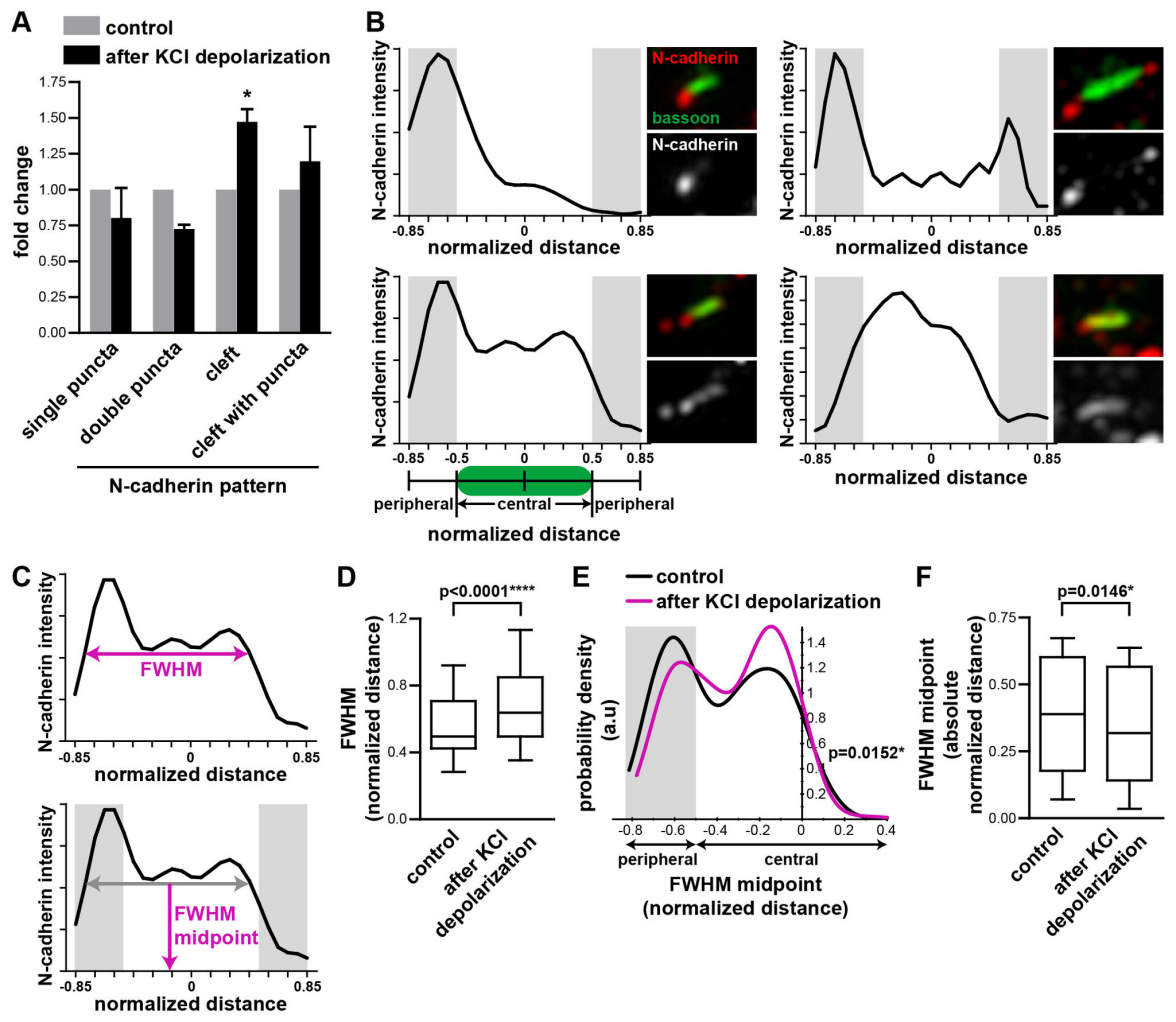
Transient synaptic stimulation relocalizes N-cadherin from the periphery to the center of the synapse. (**A**) Transient KCl depolarization followed by a rest of 15 min reduced the proportion of synapses with punctate N-cadherin adjacent to the active zone and increased the proportion of synapses with N-cadherin along the synaptic cleft. n=3 experiments, ≥120 synapses per experiment. * = p<0.05, two-way ANOVA. (**B**) Example linescans of N-cadherin fluorescence intensity along the synaptic cleft. Corresponding images are on the right of each linescan. Linescans extended beyond both ends of the active zone as delineated by the bassoon rods, and were normalised to the length of the bassoon rods. The central region was defined as the region occupied by bassoon. The peripheral region was immediately adjacent to this (shaded in gray). (**C**) Definition of the full-width at half-maximum (FWHM) and FWHM midpoint. (**D**) Box and whiskers graph of the FWHM distribution. Transient KCl depolarization increased the FWHM of the N-cadherin fluorescence peak. n≥339 synapses per condition. Mann Whitney test. (**E**) The probability density function for the distribution of the FWHM midpoint values. Transient KCl depolarization shifted the FWHM midpoint of the N-cadherin fluorescence peak from a peripheral to a central synaptic location. n≥339 synapses per condition. Mann Whitney test. (**F**) Box and whiskers graph of the absolute value of the FWHM midpoint. After transient KCl depolarization, the absolute FWHM midpoint decreased, indicating a shift towards the center of the synapse. n=3 experiments, n≥214 synapses per experiment. Mann Whitney test.

Stimulation of neurons by transient KCl depolarization followed by 15 min rest in normal media reduced the proportion of synapses with N-cadherin present as a single or double puncta adjacent to the active zone and increased the proportion of synapses with N-cadherin present along the synaptic cleft (Figure 4A), compared to control unstimulated cells. The increased proportion of synapses with N-cadherin at the synaptic cleft was also reflected by increased N-cadherin at the active zone, as measured by a significant increase in the fraction of bassoon at the synapse that colocalized with N-cadherin (Figure 3C).

To quantitate this effect in more detail, we performed linescans of the N-cadherin fluorescence intensity along 1.7 x the length of the bassoon rods, extending beyond the bassoon rods to capture N-cadherin puncta peripheral to the active zone. The linescan lengths were normalized such that the bassoon rods had a length of 1, with the linescan extending 0.35 units beyond each end. The linescans were oriented so that the majority of the fluorescence was on the left (Figure 4B). To measure the spread of N-cadherin fluorescence along the synapse, we calculated the full-width at half-maximum (FWHM) (Figure 4C) of the major peak. Although secondary peaks were sometimes present, measurement of the major peak captured where the majority of the N-cadherin fluorescence was concentrated and the general trends that were detected from visual inspection of the images. Transient KCl depolarization increased the FWHM (Figure 4D), consistent with N-cadherin being less punctate and more broadly distributed along the synaptic cleft. To measure where the bulk of the N-cadherin fluorescence was positioned, we calculated the FWHM midpoint (Figure 4C). The distribution of the FWHM midpoint values had two peaks – one peripheral to the active zone and one in the central region of the synapse (Figure 4E), representing synapses that predominantly have N-cadherin peripherally versus centrally distributed respectively. After transient KCl depolarization fewer synapses had a peripheral FWHM midpoint and more synapses had a central FWHM midpoint. To measure this change in FWHM midpoint more directly, we took the absolute value of the FWHM midpoint as the distance from the center of the synapse. After transient KCl depolarization, the absolute value of the FWHM midpoint decreased (Figure 4F), indicating a shift in N-cadherin fluorescence from the periphery to the center of the synapse. Together, our data on the N-cadherin localization pattern (Figure 4A), degree of N-cadherin colocalization with bassoon (Figure 3C), and distribution of N-cadherin fluorescence at the synapse (Figure 4D-F) support a model where after transient KCl depolarization, N-cadherin relocalizes from the periphery to the center of the synapse.

Although transient KCl depolarization changed the localization of N-cadherin relative to bassoon, it had no significant effect on the length of the bassoon rods compared to the control (Figure 3E). There was a slight (12%) decrease in the integrated N-cadherin fluorescence intensity at synapses after transient KCl depolarization (Figure 3F). However, the biological implications of this small decrease in N-cadherin at the synapse are unclear. We did not find a correlation between the integrated N-cadherin fluorescence intensity and the fraction of bassoon which overlapped with N-cadherin. This suggests that the change in the N-cadherin colocalized with bassoon that we observe under the different conditions does not correlate with the amount of N-cadherin at the synapse. Hence it is unlikely that the small decrease in N-cadherin at the synapse is directly related to the changes we see in N-cadherin localization within the synapse. Previous studies have shown that transient KCl stimulation increases N-cadherin endocytosis and reduces N-cadherin cell surface levels [22], however, this effect is only detectable 4 hr after stimulation. On the other hand, late phase LTP is associated with an increase in N-cadherin protein levels [36]. Since we observed our neurons 15 min after KCl stimulation, it is not clear if any of these mechanisms would act in this short time period.

In this study, we observed that N-cadherin is often clustered at the synapse, appearing more like spot-welds [23,24,41] that may correspond to puncta adherentia [25], than a ring-like gasket as has been previously proposed [6,26]. Hence it is unlikely that N-cadherin confines neurotransmitter diffusion at the synapse simply by acting as a molecular gasket. However, the localization of N-cadherin within the synapse could still influence neurotransmitter movement in the synaptic cleft. 

**Figure 5:**
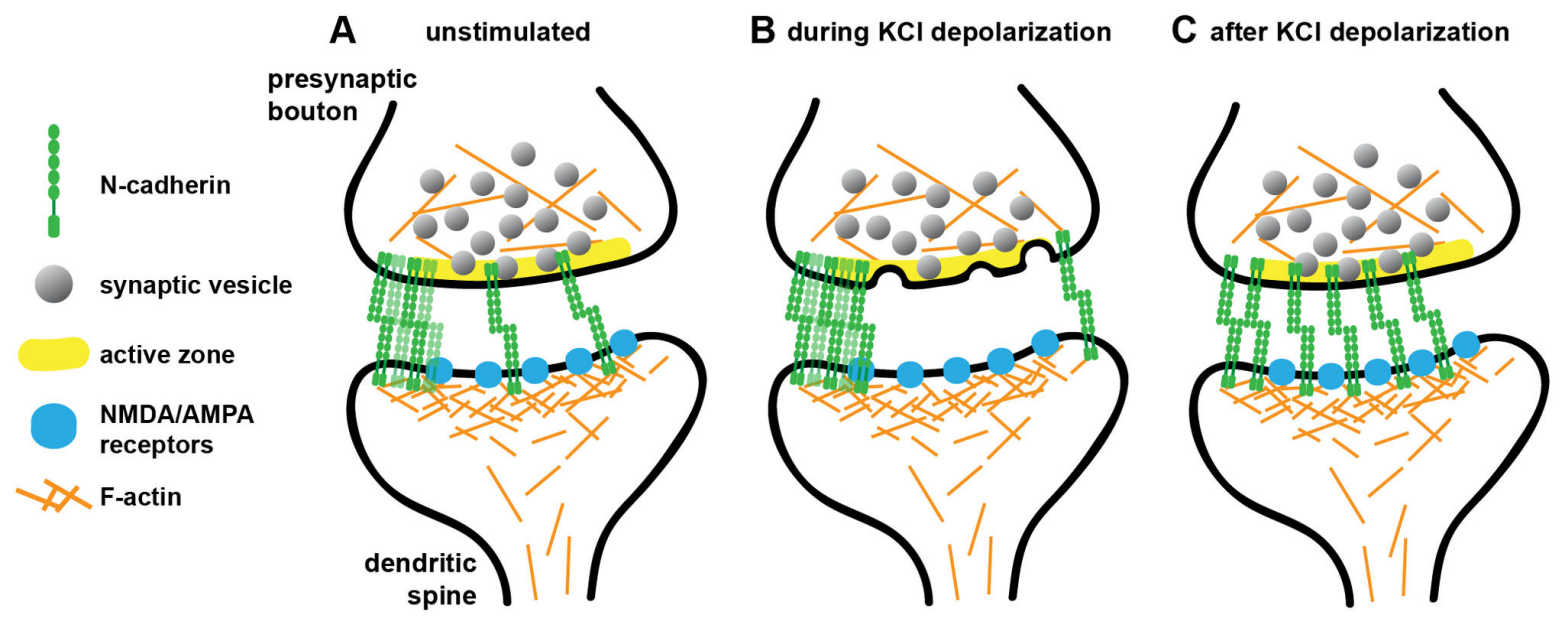
N-cadherin relocalizes to the center of the synapse after transient synaptic stimulation. (**A**) In unstimulated neurons, N-cadherin is present at synapses in a variety of distributions. It is primarily found as puncta near the edges of the active zone, but can also be present as a more uniform distribution along the synaptic cleft. (**B**) During KCl depolarization, there is less N-cadherin in the central region along the active zone, and more N-cadherin at periphery of the active zone. (**C**) After transient synaptic stimulation with KCl, N-cadherin relocalizes to form a broader central distribution along the active zone. This change in N-cadherin localization may have consequences for the increase in synaptic strength and stability following synaptic activity.

We show that in unstimulated neurons, N-cadherin is present predominantly as puncta at the periphery of the synapses, with some low amounts in the synaptic cleft (Figure 5A). During synaptic stimulation by KCl depolarization, N-cadherin redistributed to the periphery of the synapse, and was almost entirely absent from the active zone region (Figure 5B). Part of the reason for this may be increased synaptic vesicle fusion at the active zone which adds membrane without N-cadherin to the active zone. After transient synaptic stimulation N-cadherin moves back from the periphery where it was present as puncta, to the centre of the synapse where it is more homogenously localized (Figure 5C). This last state represents a different N-cadherin localization pattern compared to unstimulated synapses, indicating that transient synaptic stimulation has an impact on N-cadherin localization beyond the time period of the stimulation. 

This redistribution of N-cadherin after synaptic stimulation may be due to changes in β-catenin localization. Transient KCl depolarization causes a redistribution of β-catenin from dendritic shafts to dendritic spines. This is associated with an increased association between β-catenin and cadherins [40]. Therefore, increased β-catenin in dendritic spines after transient synaptic stimulation could drive the preferential relocalization of N-cadherin to the central region of the synaptic cleft that we observe. We did not find large differences in the amount of N-cadherin at the synapse either during or after transient KCl stimulation, suggesting that the changes we observed are predominantly due to a relocalization of N-cadherin at the synapse. N-cadherin protein levels are elevated in late phase LTP [17], which presumably is subsequent to the short term changes described in this work. 

This relocalization of N-cadherin could affect the diffusion of neurotransmitter receptors in the postsynaptic membrane through macromolecular crowding, an effect where the presence of large numbers of high-molecular weight proteins significantly restricts protein diffusion [42]. Constraining the lateral diffusion of neurotransmitter receptors could confine them more centrally within the synapse. Indeed, the diffusion of AMPARs within the synapse is ~5 fold lower than in the extra-synaptic membrane [43], suggesting that proteins in the synaptic compartment can modulate diffusion rates and retain AMPARs within the PSD.

Besides influencing movement of neurotransmitter receptors at the synapse through macromolecular crowding, the relocalization of N-cadherin at the synapse may also have direct effects on AMPARs. N-cadherin directly interacts extracellularly with the GluR2 subunit of AMPAR [13], and indirectly with AMPARs through a complex containing δ-catenin (neural plakophilin-related arm protein) and AMPAR binding protein (ABP)/glutamate receptor interacting protein (GRIP) [44]. These interactions can recruit and stabilize AMPARs at the synapse [12,13,44]. Thus changes in N-cadherin localization may result in changes in the stability and localization of AMPARs. AMPAR trafficking and regulation is a key factor that regulates synaptic strength [45], and the local density of AMPARs within the PSD may influence the efficiency of synaptic transmission [46]. Interestingly, AMPARs are not always randomly distributed within the PSD, but can be situated more peripherally than centrally in the PSD [47,48], and may have a spectrum of localization patterns from more central to more peripheral distributions [31]. These could be regulated in part by changes in N-cadherin localization within synapses.

N-cadherin also displays increased trypsin resistance and dimerization after transient synaptic stimulation, indicators of enhanced N-cadherin-mediated cellular adhesion [20]. The enhanced protease resistance may be due to the relocalization of N-cadherin from the peripheral to the central regions of the synapse where it is more protected. Furthermore, increases in the amount of N-cadherin at a synapse can stabilize synapses [10,49], so perhaps the actual localization and binding strength of N-cadherin could affect synapse stability as well. Our findings that transient synaptic activity changes the localization of N-cadherin within synapses adds to the exquisite body of knowledge on how N-cadherin may mediate activity-dependent changes at the synapse.

## Materials and Methods

### Ethics statement and hippocampal neuron culture

All animal work was approved by The MNI Animal Care Committee of Mcgill University (Animal Welfare Assurance #A5006-01) and performed in accordance with The Canadian Council on Animal Care Guidelines. Pregnant Sprague Dawley rats (Charles River, St.-Constant, Canada) were euthanized by carbon dioxide inhalation. Hippocampi were dissected from E17-18 rat embryos [50]. Hippocampal neurons were cultured in Neurobasal medium supplemented with L-glutamine and B27 (Invitrogen) and used at 11 DIV or 17-20 DIV. 

### Depolarization of neurons

Neurons were depolarized with 30-50 mM KCl for 3-4 min in conditioned culture medium, as previously described [20]. This range of KCl had similar effects on neuronal depolarization, consistent with previous studies [20]. For the fixation of cells during KCl depolarization, the neurons were then immediately fixed in 4% paraformaldehyde or -20°C methanol. Otherwise, for neurons that were transiently depolarized and then returned to normal media, the depolarization media was replaced with conditioned media and the neurons returned to the cell culture incubator for 15 minutes, before being fixed in 4% paraformaldehyde or -20°C methanol.

### Immunostaining

Immunostaining was performed as described previously [20]. Neurons were fixed in 4% paraformaldehyde for 15 min at room temperature, or in -20 °C methanol for 10 min. The primary antibody was incubated overnight at 4°C with the sample in PBS with 0.5% normal goat serum and 0.1% triton X-100, and standard immunostaining procedures followed. The following primary antibodies were used: mouse anti-bassoon (SAP7F407, Assay Designs, USA), guinea pig anti-bassoon (141-004, raised against the 330 C-terminal amino acids of rat bassoon, Synaptic Systems, Germany), mouse anti-PSD95 (MA1-045, Thermo Scientific, USA), rabbit anti-synaptophysin (08-0130, Invitrogen), guinea pig anti-vGlut1 (AB5905, Millipore, USA), and rabbit anti-N-cadherin raised against the intracellular domain of N-cadherin fused to glutathione-S-transferase (GST) [20]. The rabbit-anti-N-cadherin antibody was affinity purified using the same intracellular N-cadherin domain [20].

### Structured illumination microscopy

SIM was performed as previously described [51] on a 3D structured illumination microscope (OMX v3, Applied Precision) equipped with 405, 488 and 592.5 nm diode lasers, electron multiplying CCD (charge-coupled device) cameras (Cascade II 512×512, Photometrics), and a 100×/1.40 NA planApochromat oil-immersion objective (Olympus). Image reconstruction and processing was performed as described [51] except that alignment of the different channels with Acapella software was performed on 16-bit images prior to making a maximum intensity projection.

### Colocalization measurements

To measure the proportion of the bassoon signal colocalized with either PSD95 or vGlut1, and vice versa, we used maximum intensity projected and aligned 3D-SIM images for analysis with the ImageJ (National Institutes of Health, USA) plugin JACoP [30] (http://imagejdocu.tudor.lu/doku.php?id=plugin:analysis:jacop_2.0:just_another_colocalization_plugin:start). The Manders’ coefficient was calculated from thresholded images.

To measure the fraction of each bassoon rod which overlapped with N-cadherin (colocalized), we used reconstructed, maximum intensity projected, and aligned 3D-SIM images for analysis with MATLAB (scripts can be provided upon request). For each image set, the bassoon rods were detected using minimum signal-to-noise (10x), size and morphology thresholds to generate masks. Each bassoon mask was dilated by 5 pixels and subsequently used to search for N-cadherin staining in this region. We then quantitated the fraction of pixels corresponding to N-cadherin staining that overlapped with a given bassoon mask. We also measured the integrated fluorescence intensity of the same N-cadherin staining associated with each bassoon mask.

### Volume measurements

The volume of vGlut1 and synaptophysin puncta were measured with Imaris (Bitplane, Zurich, Switzerland).

### Linescans of fluorescence intensity

To perform the linescans of N-cadherin fluorescence intensity, bassoon puncta were identified by user-defined thresholding of bassoon images followed by identification of rod-shaped bassoon puncta with a minimum length of 0.25 µm that correspond to synapses. The position of the edges of these puncta were identified as 2D polygons; the major axis of this polygon was identified as the first eigenvector of the covariance matrix of the (x, y) coordinates of the outline. These polygons were axis-aligned and a simple coordinate system was defined along the major and minor axes such that (-1,-1) corresponded to the bottom-left of the bounding box of the active zone, and (+1,+1) corresponded to the top-right. “Linescans” measuring average fluorescence in the N-cadherin channel at 25 evenly spaced positions along the major axis from -1.35 to +1.35 in this coordinate system (thus extending beyond the edges of the bassoon rod) were performed, with averaging along the minor axis from -1.1 to +1.1. Linescans were flipped where necessary such that the greatest fluorescence intensity was on the left half. Linescans where the maximum value was below 2x the background levels were excluded from analysis. The “full width at half maximum” of these linescans was calculated by identifying the position (in the relative coordinate system along the major axis) of the maximum value, and then identifying the first positions to the left and right of that maximum which fell below half of that value. The “FWHM midpoint” was defined as the average of the left and the right positions at which the fluorescence fell below half the maximum value.

### Graphs and statistics

Statistical analyses were performed with GraphPad Prism 5 for Windows (La Jolla, CA). The probability density functions representing frequency distributions were calculated using the Celltool software suite [52], with the “plot_distribution” tool which uses Gaussian kernel density estimation http://pantheon.yale.edu/~zp2/Celltool/. The probability density function describes the relative likelihood for the variable of interest (fraction of bassoon with N-cadherin overlap; FWHM midpoint) to take on a given value.

## Supporting Information

Figure S1 **Bassoon puncta volume is the same under different co-immunostaining conditions.** 17-20 DIV hippocampal neurons were immunostained for the active zone protein bassoon and the post-synaptic density protein PSD95 (bassoon/PSD95), or bassoon and the synaptic vesicle protein vGlut1 (bassoon/vGlut1). The individual bassoon puncta volumes are the same under the two co-immunostaining conditions. Mann Whitney test, p=0.2401, ≥ 4 fields of view per condition, ≥509 bassoon puncta analyzed per condition.
(TIF)

## References

[1] Redies C (2000) Cadherins in the central nervous system. Prog Neurobiol 61: 611–648. doi:10.1016/S0301-0082(99)00070-2. PubMed: 10775799. 10775799

[2] Takeichi M (2007) The cadherin superfamily in neuronal connections and interactions. Nat Rev Neurosci 8: 11–20. doi:10.1038/nrn2043. PubMed: 17133224. 17133224

[3] Brasch J, Harrison OJ, Honig B, Shapiro L (2012) Thinking outside the cell: how cadherins drive adhesion. Trends Cell Biol 22: 299–310. doi:10.1016/j.tcb.2012.03.004. PubMed: 22555008. 22555008 PMC3385655

[4] Arikkath J, Reichardt LF (2008) Cadherins and catenins at synapses: roles in synaptogenesis and synaptic plasticity. Trends Neurosci 31: 487–494. doi:10.1016/j.tins.2008.07.001. PubMed: 18684518. 18684518 PMC2623250

[5] Benson DL, Tanaka H (1998) N-cadherin redistribution during synaptogenesis in hippocampal neurons. J Neurosci 18: 6892–6904. PubMed: 9712659. 9712659 10.1523/JNEUROSCI.18-17-06892.1998 PMC6792987

[6] Fannon AM, Colman DR (1996) A model for central synaptic junctional complex formation based on the differential adhesive specificities of the cadherins. Neuron 17: 423–434. doi:10.1016/S0896-6273(00)80175-0. PubMed: 8816706. 8816706

[7] Stan A, Pielarski KN, Brigadski T, Wittenmayer N, Fedorchenko O et al. (2010) Essential cooperation of N-cadherin and neuroligin-1 in the transsynaptic control of vesicle accumulation. Proc Natl Acad Sci USA 107: 11116–11121. doi:10.1073/pnas.0914233107. PubMed: 20534458. 20534458 PMC2890764

[8] Togashi H, Abe K, Mizoguchi A, Takaoka K, Chisaka O et al. (2002) Cadherin regulates dendritic spine morphogenesis. Neuron 35: 77–89. doi:10.1016/S0896-6273(02)00748-1. PubMed: 12123610. 12123610

[9] Bamji SX, Shimazu K, Kimes N, Huelsken J, Birchmeier W et al. (2003) Role of [beta]-catenin in synaptic vesicle localization and presynaptic assembly. Neuron 40: 719–731. doi:10.1016/S0896-6273(03)00718-9. PubMed: 14622577. 14622577 PMC2757419

[10] Mendez P, De Roo M, Poglia L, Klauser P, Muller D (2010) N-cadherin mediates plasticity-induced long-term spine stabilization. J Cell Biol 189: 589 –600. doi:10.1083/jcb.201003007. PubMed: 20440002. 20440002 PMC2867305

[11] Xie Z, Photowala H, Cahill ME, Srivastava DP, Woolfrey KM et al. (2008) Coordination of synaptic adhesion with dendritic spine remodeling by AF-6 and Kalirin-7. J Neurosci 28: 6079–6091. doi:10.1523/JNEUROSCI.1170-08.2008. PubMed: 18550750. 18550750 PMC2727754

[12] Nuriya M, Huganir RL (2006) Regulation of AMPA receptor trafficking by N-cadherin. J Neurochem 97: 652–661. doi:10.1111/j.1471-4159.2006.03740.x. PubMed: 16515543. 16515543

[13] Saglietti L, Dequidt C, Kamieniarz K, Rousset M-C, Valnegri P et al. (2007) Extracellular interactions between GluR2 and N-cadherin in spine regulation. Neuron 54: 461–477. doi:10.1016/j.neuron.2007.04.012. PubMed: 17481398. 17481398

[14] Bozdagi O, Wang XB, Nikitczuk JS, Anderson TR, Bloss EB et al. (2010) Persistence of coordinated long-term potentiation and dendritic spine enlargement at mature hippocampal CA1 synapses requires N-cadherin. J Neurosci 30: 9984–9989. doi:10.1523/JNEUROSCI.1223-10.2010. PubMed: 20668183. 20668183 PMC2921177

[15] Okamura K, Tanaka H, Yagita Y, Saeki Y, Taguchi A et al. (2004) Cadherin activity is required for activity-induced spine remodeling. J Cell Biol 167: 961–972. doi:10.1083/jcb.200406030. PubMed: 15569714. 15569714 PMC2172468

[16] Tang L, Hung CP, Schuman EM (1998) A role for the cadherin family of cell adhesion molecules in hippocampal long-term potentiation. Neuron 20: 1165–1175. doi:10.1016/S0896-6273(00)80497-3. PubMed: 9655504. 9655504

[17] Bozdagi O, Shan W, Tanaka H, Benson DL, Huntley GW (2000) Increasing numbers of synaptic puncta during late-phase LTP: N-cadherin is synthesized, recruited to synaptic sites, and required for potentiation. Neuron 28: 245–259. doi:10.1016/S0896-6273(00)00100-8. PubMed: 11086998. 11086998

[18] Vitureira N, Letellier M, White IJ, Goda Y (2012) Differential control of presynaptic efficacy by postsynaptic N-cadherin and β-catenin. Nat Neurosci 15: 81–89. doi:10.1038/nn.2995. PubMed: 22138644. PMC3245860 22138644

[19] Jüngling K, Eulenburg V, Moore R, Kemler R, Lessmann V et al. (2006) N-Cadherin transsynaptically regulates short-term plasticity at glutamatergic synapses in embryonic stem cell-derived neurons. J Neurosci 26: 6968–6978. doi:10.1523/JNEUROSCI.1013-06.2006. PubMed: 16807326. 16807326 PMC6673917

[20] Tanaka H, Shan W, Phillips GR, Arndt K, Bozdagi O et al. (2000) Molecular modification of N-cadherin in response to synaptic activity. Neuron 25: 93–107. doi:10.1016/S0896-6273(00)80874-0. PubMed: 10707975. 10707975

[21] Tai C-Y, Mysore SP, Chiu C, Schuman EM (2007) Activity-regulated N-cadherin endocytosis. Neuron 54: 771–785. doi:10.1016/j.neuron.2007.05.013. PubMed: 17553425. 17553425

[22] Yasuda S, Tanaka H, Sugiura H, Okamura K, Sakaguchi T et al. (2007) Activity-induced protocadherin arcadlin regulates dendritic spine number by triggering N-cadherin endocytosis via TAO2[beta] and p38 MAP kinases. Neuron 56: 456–471. doi:10.1016/j.neuron.2007.08.020. PubMed: 17988630. 17988630 PMC2424284

[23] Elste AM, Benson DL (2006) Structural basis for developmentally regulated changes in cadherin function at synapses. J Comp Neurol 495: 324–335. doi:10.1002/cne.20876. PubMed: 16440298. 16440298

[24] Uchida N, Honjo Y, Johnson KR, Wheelock MJ, Takeichi M (1996) The catenin/cadherin adhesion system is localized in synaptic junctions bordering transmitter release zones. J Cell Biol 135: 767–779. doi:10.1083/jcb.135.3.767. PubMed: 8909549. 8909549 PMC2121068

[25] Spacek J, Harris KM (1998) Three-dimensional organization of cell adhesion junctions at synapses and dendritic spines in area CA1 of the rat hippocampus. J Comp Neurol 393: 58–68. doi:10.1002/(SICI)1096-9861(19980330)393:1<58::AID-CNE6>3.0.CO;2-P. PubMed: 9520101. 9520101 10.1002/(sici)1096-9861(19980330)393:1<58::aid-cne6>3.0.co;2-p

[26] Dustin ML, Colman DR (2002) Neural and immunological synaptic relations. Science 298: 785–789. doi:10.1126/science.1076386. PubMed: 12399580. 12399580

[27] Gustafsson MGL (2000) Surpassing the lateral resolution limit by a factor of two using structured illumination microscopy. J Microsc 198: 82–87. doi:10.1046/j.1365-2818.2000.00710.x. PubMed: 10810003. 10810003

[28] Gustafsson MGL, Shao L, Carlton PM, Wang CJR, Golubovskaya IN et al. (2008) Three-dimensional resolution doubling in wide-field fluorescence microscopy by structured illumination. Biophys J 94: 4957–4970. doi:10.1529/biophysj.107.120345. PubMed: 18326650. 18326650 PMC2397368

[29] Schermelleh L, Carlton PM, Haase S, Shao L, Winoto L et al. (2008) Subdiffraction multicolor imaging of the nuclear periphery with 3D structured illumination microscopy. Science 320: 1332–1336. doi:10.1126/science.1156947. PubMed: 18535242. 18535242 PMC2916659

[30] Bolte S, Cordelières FP (2006) A guided tour into subcellular colocalization analysis in light microscopy. J Microsc 224: 213–232. doi:10.1111/j.1365-2818.2006.01706.x. PubMed: 17210054. 17210054

[31] Dani A, Huang B, Bergan J, Dulac C, Zhuang X (2010) Superresolution imaging of chemical synapses in the brain. Neuron 68: 843–856. doi:10.1016/j.neuron.2010.11.021. PubMed: 21144999. 21144999 PMC3057101

[32] Siksou L, Rostaing P, Lechaire J-P, Boudier T, Ohtsuka T et al. (2007) Three-dimensional architecture of presynaptic terminal cytomatrix. J Neurosci 27: 6868–6877. doi:10.1523/JNEUROSCI.1773-07.2007. PubMed: 17596435. 17596435 PMC6672225

[33] Triller A, Korn H (1985) Activity-dependent deformations of presynaptic grids at central synapses. J Neurocytol 14: 177–192. doi:10.1007/BF01258446. PubMed: 4045503. 4045503

[34] Jontes JD, Emond MR, Smith SJ (2004) In vivo trafficking and targeting of N-cadherin to nascent presynaptic terminals. J Neurosci 24: 9027–9034. doi:10.1523/JNEUROSCI.5399-04.2004. PubMed: 15483121. 15483121 PMC6730076

[35] Phillips GR, Huang JK, Wang Y, Tanaka H, Shapiro L et al. (2001) The presynaptic particle web: Ultrastructure, composition, dissolution, and reconstitution. Neuron 32: 63–77. doi:10.1016/S0896-6273(01)00450-0. PubMed: 11604139. 11604139

[36] Bozdagi O, Valcin M, Poskanzer K, Tanaka H, Benson DL (2004) Temporally distinct demands for classic cadherins in synapse formation and maturation. Mol Cell Neurosci 27: 509–521. doi:10.1016/j.mcn.2004.08.008. PubMed: 15555928. 15555928 PMC2910522

[37] Zhang W, Benson DL (2001) Stages of synapse development defined by dependence on F-actin. J Neurosci 21: 5169–5181. PubMed: 11438592. 11438592 10.1523/JNEUROSCI.21-14-05169.2001 PMC6762826

[38] Rubio ME, Curcio C, Chauvet N, Brusés JL (2005) Assembly of the N-cadherin complex during synapse formation involves uncoupling of p120-catenin and association with presenilin 1. Mol Cell Neurosci 30: 118–130. doi:10.1016/j.mcn.2005.06.005. PubMed: 16046145. 16046145

[39] Sigrist SJ, Sabatini BL (2012) Optical super-resolution microscopy in neurobiology. Curr Opin Neurobiol 22: 86–93. doi:10.1016/j.conb.2011.10.014. PubMed: 22051692. 22051692

[40] Murase S, Mosser E, Schuman EM (2002) Depolarization drives [beta]-catenin into neuronal spines promoting changes in synaptic structure and function. Neuron 35: 91–105. doi:10.1016/S0896-6273(02)00764-X. PubMed: 12123611. 12123611

[41] Colman DR (1997) Neurites, synapses, and cadherins reconciled. Mol Cell Neurosci 10: 1–6. doi:10.1006/mcne.1997.0648. PubMed: 9361283. 9361283

[42] Santamaria F, Gonzalez J, Augustine GJ, Raghavachari S (2010) Quantifying the effects of elastic collisions and non-covalent binding on glutamate receptor trafficking in the post-synaptic density. PLOS Comput Biol 6: e1000780. doi:10.1371/journal.pcbi.1000780. PubMed: 20485563. 20485563 PMC2869312

[43] Tardin C, Cognet L, Bats C, Lounis B, Choquet D (2003) Direct imaging of lateral movements of AMPA receptors inside synapses. EMBO J 22: 4656–4665. doi:10.1093/emboj/cdg463. PubMed: 12970178. 12970178 PMC212729

[44] Silverman JB, Restituito S, Lu W, Lee-Edwards L, Khatri L et al. (2007) Synaptic anchorage of AMPA receptors by cadherins through neural plakophilin-related arm protein–AMPA receptor-binding protein complexes. J Neurosci 27: 8505 –8516. doi:10.1523/JNEUROSCI.1395-07.2007. PubMed: 17687028. 17687028 PMC6672939

[45] Bredt DS, Nicoll RA (2003) AMPA receptor trafficking at excitatory synapses. Neuron 40: 361–379. doi:10.1016/S0896-6273(03)00640-8. PubMed: 14556714. 14556714

[46] MacGillavry HD, Kerr JM, Blanpied TA (2011) Lateral organization of the postsynaptic density. Mol Cell Neurosci 48: 321–331. doi:10.1016/j.mcn.2011.09.001. PubMed: 21920440. 21920440 PMC3216044

[47] Chen X, Winters C, Azzam R, Li X, Galbraith JA et al. (2008) Organization of the core structure of the postsynaptic density. Proc Natl Acad Sci USA 105: 4453–4458. doi:10.1073/pnas.0800897105. PubMed: 18326622. 18326622 PMC2393784

[48] Kharazia VN, Weinberg RJ (1997) Tangential synaptic distribution of NMDA and AMPA receptors in rat neocortex. Neurosci Lett 238: 41–44. doi:10.1016/S0304-3940(97)00846-X. PubMed: 9464650. 9464650

[49] Latefi NS, Pedraza L, Schohl A, Li Z, Ruthazer ES (2009) N-cadherin prodomain cleavage regulates synapse formation in vivo. Dev Neurobiol 69: 518–529. doi:10.1002/dneu.20718. PubMed: 19365814. 19365814 PMC2744366

[50] Kaech S, Banker G (2006) Culturing hippocampal neurons. Nat Protoc 1: 2406–2415. doi:10.1038/nprot.2006.356. PubMed: 17406484. 17406484

[51] Lawo S, Hasegan M, Gupta GD, Pelletier L (2012) Subdiffraction imaging of centrosomes reveals higher-order organizational features of pericentriolar material. Nat Cell Biol 14: 1148–1158. doi:10.1038/ncb2591. PubMed: 23086237. 23086237

[52] Pincus Z, Theriot JA (2007) Comparison of quantitative methods for cell-shape analysis. J Microsc 227: 140–156. doi:10.1111/j.1365-2818.2007.01799.x. PubMed: 17845709. 17845709

