# Supplementary figures and images for: N-Cadherin Relocalizes from the Periphery to the Center of the Synapse after Transient Synaptic Stimulation in Hippocampal Neurons
Source: PLoS One. 2013 Nov 1;8(11):e79679. doi: 10.1371/journal.pone.0079679 (PMC3815108; doi:10.1371/journal.pone.0079679)

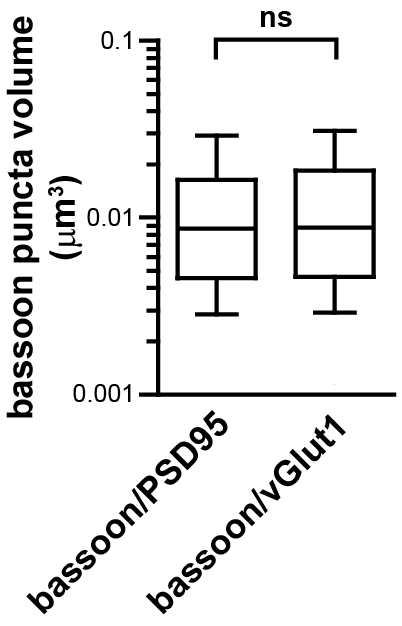

Supplement: Figure S1 — Bassoon puncta volume is the same under different co-immunostaining conditions. 17-20 DIV hippocampal neurons were immunostained for the active zone protein bassoon and the post-synaptic density protein PSD95 (bassoon/PSD95), or bassoon and the synaptic vesicle protein vGlut1 (bassoon/vGlut1). The individual bassoon puncta volumes are the same under the two co-immunostaining conditions. Mann Whitney test, p=0.2401, ≥ 4 fields of view per condition, ≥509 bassoon puncta analyzed per condition. (TIF) [file pone.0079679.s001.tif]
